# Phenological tracking associated with increased salmon consumption by brown bears

**DOI:** 10.1038/s41598-018-29425-3

**Published:** 2018-07-20

**Authors:** William W. Deacy, Joy A. Erlenbach, William B. Leacock, Jack A. Stanford, Charles T. Robbins, Jonathan B. Armstrong

**Affiliations:** 1 Department of Fisheries and Wildlife, Oregon State University, Corvallis, OR USA; 2 Flathead Lake Biological Station, University of Montana, Missoula, MT USA; 3 School of the Environment, Washington State University, Pullman, WA USA; 4 Kodiak National Wildlife Refuge, United States Fish and Wildlife Service, Kodiak, AK USA; 5 School of Biological Sciences, Washington State University, Pullman, WA USA

## Abstract

There is growing interest in the ecological significance of phenological diversity, particularly in how spatially variable resource phenologies (i.e. resource waves) prolong foraging opportunities for mobile consumers. While there is accumulating evidence of consumers moving across landscapes to surf resource waves, there is little data quantifying how phenological tracking influences resource consumption due to the challenge of documenting all the components of this ecological phenomenon (i.e., phenological variation, consumer movement, resource consumption, and consumer fitness). We examined the space use of GPS collared female brown bears to quantify the exploitation of a salmon resource wave by individual bears. We then estimated salmon consumption levels in the same individuals using stable isotope and mercury analyses of hair. We found strong positive relationships between time spent on salmon streams and percent salmon in assimilated diets (R^2^ = 0.70) and salmon mass consumed (R^2^ = 0.49). Salmon abundance varied 2.5-fold between study years, yet accounting for salmon abundance did not improve salmon consumption models. Resource abundance generally is viewed as the key variable controlling consumption levels and food web dynamics. However, our results suggest that in intact watersheds of coastal Alaska with abundant salmon runs, interannual variation in salmon abundance likely has less effect on salmon consumption than individual variation in bear foraging behavior. The results complement previous work to demonstrate the importance of phenological variation on bear foraging behavior and fitness.

## Introduction

Spatial variation in the timing of food resources generates resource waves in which mobile consumers can exploit resource pulses as they propagate across landscapes. This allows consumers to feed for much longer at larger spatial extents^1^. Animals that move to track spatial variation in resource phenology (i.e., surf the resource wave) thereby prolong their access to locally ephemeral foods. Resource waves occur throughout nature and a wide variety of consumers surf them. Early examples focused on herbivores exploiting the “green waves” of plant development^2–4^. More recent examples have documented phenological tracking by carnivorous fish^5^, omnivorous mammals^6,7^, and herbivorous^8^ and piscivorous birds^9^.

But, the degree to which consumers benefit from following resource waves, especially in terms of consumption, growth and fitness, is poorly documented. A handful of studies have quantified fitness proxies for mobile consumers under contrasting levels of phenological variation expressed among locations^10^ or across time^11,12^. However, these studies lacked behavioral data needed to quantify individual tracking, so they do not provide a direct link between phenological tracking and fitness benefits. Two studies have analyzed consumer fitness proxies as a function of phenological tracking behavior, but both in the context of partial migration where individuals were classified as resource wave surfers or non-surfers^4,5^. Recent studies of individual movements have revealed a continuum of tracking behaviors ranging from no tracking to near perfect tracking^7,13^. There is a strong need for research that quantifies how this variation in individual behavior affects levels of resource consumption, a variable which is closely linked to fitness.

**Figure 1:**
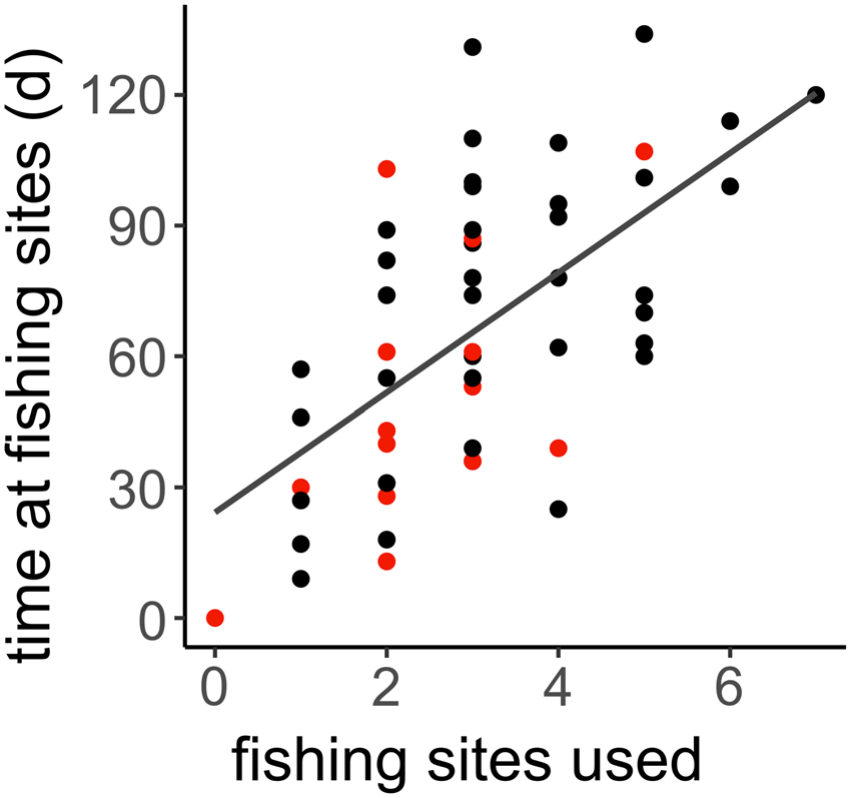
Time spent at fishing sites as a function of fishing sites used by bears. The red points (n = 18) indicate bears with associated diet data (displayed in Fig. 2). The line shows a simple linear regression (y = 13.74x + 24.25, n = 52, t = 5.46, p < 0.0001). Modified from Deacy *et al*. (2016).

Recent research established that brown bears (Ursus arctos) prolong their access to spawning sockeye salmon (Oncorhynchus nerka) by tracking consistent patterns of landscape-level variation in spawning phenology^7,14^. Bears in Kodiak (Alaska, USA), moved among spawning sites in the order required to track spatial variation in spawn timing. Although individual salmon populations were only available for ~40 days, bears that visited the most spawning sites fed on salmon for as many as 120 days (Fig. 1), three times longer than would be possible if a wave did not occur or if the bears did not track it. However, bears that fish for salmon the longest do not necessarily consume the most salmon; capture efficiency varies markedly across space, time, and among bears^15,16^, so it remains unknown whether foraging duration translates into total salmon consumption. The purpose of this study is to quantify the relationship between foraging duration and diets as a first step towards understanding how resource waves affect individual fitness. We quantified assimilated diets (% of carbon and nitrogen assimilated by bears that came from sockeye salmon, plant matter, and black-tailed deer (*Odocoileus hemionus columbianus*)) and salmon consumption (kg consumed) of individual GPS-tracked female brown bears that spent varying amounts of time foraging for salmon. We used time on streams as a proxy for foraging duration, based on strong empirical evidence showing that bears are rarely near streams when salmon are absent^7^. The study was conducted in a ~1,000,000 km^2^ area in the vicinity of Karluk Lake in southwestern Kodiak Island, where salmon enter a large suite of spawning streams as a classic resource wave and are utilized by abundant, large brown bears^7,14^.

## Results

**Figure 2:**
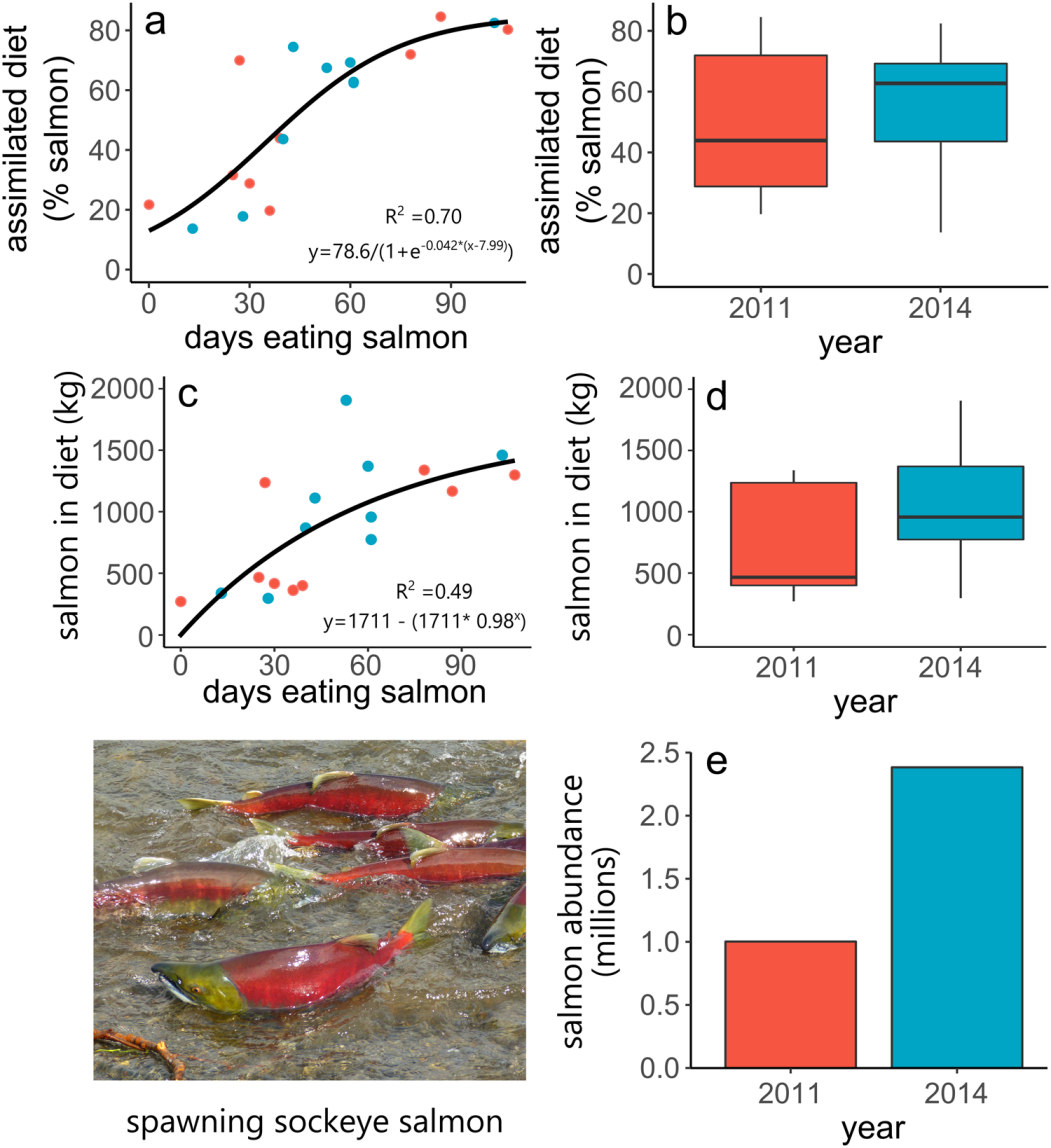
(**a**) Estimated % salmon in the assimilated diets of 18 bears. Points show the median of the posterior distributions resulting from MixSIAR models for each bear; and (**c**) kg salmon in bear diets as functions of foraging duration; (**b**) estimated % salmon in the assimilated diets and (**d**) kg of salmon in bear diets by year. At α = 0.05, % salmon in assimilated diets did not significantly differ between years (t-test; p = 0.08). However, bears consumed more kg of salmon in 2014 than 2011 (t-test; p < 0.01). **e**) total annual salmon escapement (all species) in the Karluk watershed, Alaska. In all panels, red and blue indicate data from 2011 and 2014, respectively.

Bears consumed an average of 1099 kg of salmon (n = 33; SD = 560 kg), which represented 64.5% of the assimilated diet (median; n = 33; SD = 6.2%) (Table 1, Supplementary Table 1, Supplementary Figure 1). Individuals fished at an average of 2.5 salmon spawning sites (mean; n = 18; SD = 1.2) for 41.5 days (median due to slightly skewed distribution; n = 18; SD = 29.6 days)^7^. As in a previous study^7^, time spent at fishing sites increased with the number of fishing sites used by bears (n = 18, t = 2.51, p < 0.05, Fig. 1), suggesting that bears were surfing salmon resource waves as a strategy to increase their access to salmon. We found strong relationships between foraging duration and both assimilated diet composition and salmon consumption. All three model forms (sigmoidal, simple linear, saturating) performed well (∆AICc <2)^17^, but a saturating model fit the salmon consumption data best and a sigmoidal model fit the assimilated diet data best (Supplementary Table S2). The percent salmon in assimilated bear diets reached an asymptote of ~79% when bears had fed for ~100 days (Fig. 2a). Salmon consumption reached a predicted maximum of ~1500 kg when bears had fed for ~100 days (Fig. 2c).

**Table 1:** Assimilated dietary contribution estimates from a MixSIAR model for brown bears on Kodiak Island, Alaska, 2011 and 2014.

	Mean	SD	Median	95% CI	Range
Deer	9.4	7.1	7.8	0.7–26.8	1.8, 10.1
Plant matter	26.2	7.2	26.3	12.2–40.0	3.8, 78.0
Salmon	64.4	6.2	64.5	52.0–76.0	13.3, 93.5

Models had animal identification number as a random effect and process error. Mean, 1 SD, Median, and 95% CI (credible interval) are % estimates from the population-level model. Range denotes the range of median % estimates among individual bears.

Although salmon escapement (salmon remaining after commercial and sport harvest) was higher in 2014 than in 2011 (2.4 vs. 1.0 million, Fig. 2e), a multiple regression model indicated that variation in kg salmon consumed was best explained by duration of foraging, rather than total salmon abundance (duration: t = 3.81, p < 0.01; salmon abundance: t = 0.94, p = 0.36). The mass of salmon consumed was 51% higher in 2014 than in 2011(t = −2.51, p = 0.017, Fig. 2d). Overall, individual variation in the duration of foraging had the largest effect on salmon consumption, and salmon consumption was 51% higher during a year with 140% more salmon.

**Figure 3:**
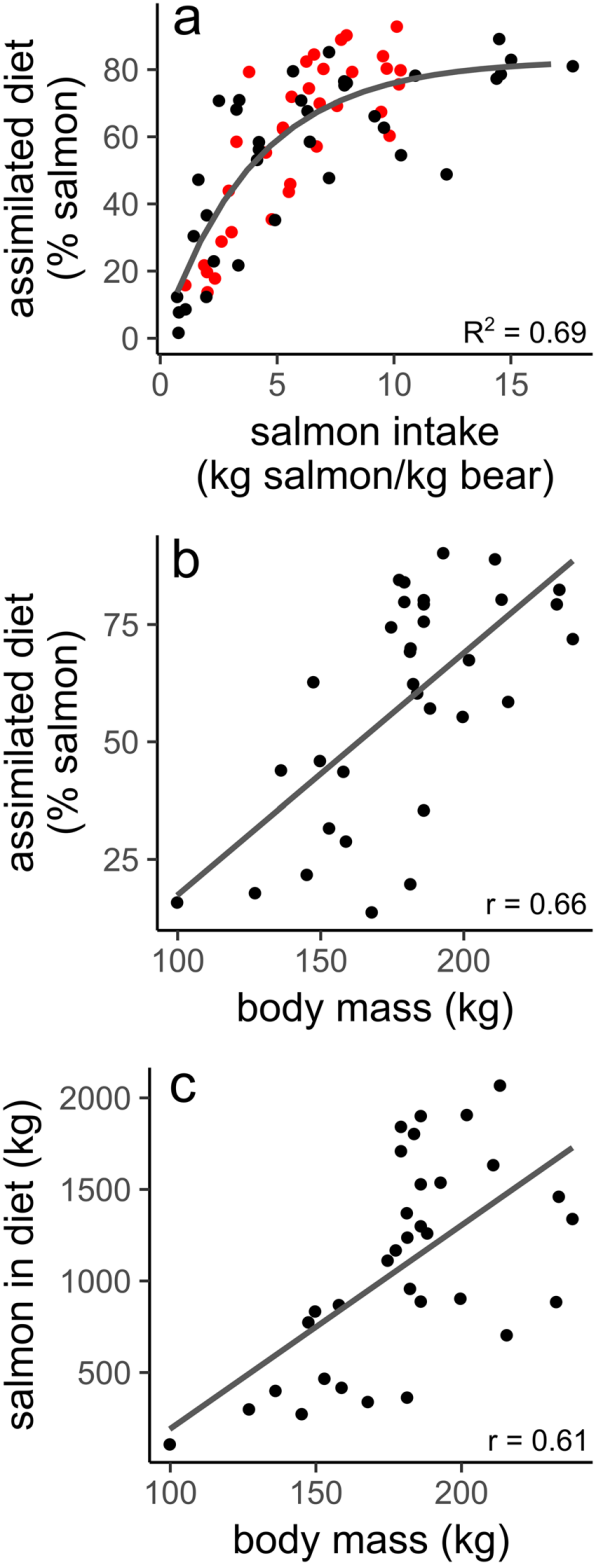
(**a**) % salmon in assimilated diet as a function of salmon intake (kg salmon/kg bear). Line shows a saturating model (y = 82.89−(82.89*(0.779^x^)). Data from current study in red (n = 33) and previously published data from other southwest Kodiak female brown bears in black (n = 36)^29^; (**b**) Correlation between % salmon in assimilated diet and body mass (n = 33); (**c**) Correlation between salmon consumption (kg) and body mass (n = 33).

Bear diets did not vary with individual reproductive status (i.e., with (n = 11) or without (n = 22) cubs) (two sample t-test: % salmon in assimilated diet: t = −0.712 p = 0.48, n = 33; salmon consumption: t = −0.24, p = 0.82, n = 33). However, our small sample sizes prevented a robust assessment of this topic. Bear diets did vary with bear mass (linear regression; assimilated diet composition, t = 4.86, p < 0.0001, n = 33; consumption, t = 4.07, p < 0.001, n = 33) as larger bears consumed more salmon as a percentage of assimilated diets (Fig. 3b) and by salmon mass consumed (Fig. 3c).

## Discussion

Salmon spawn at different times in different habitats in response to local thermal regimes^7,18^. Previous work has shown that bears prolong their access to salmon by moving in sync with the predictable pattern of salmon availability^7,14^, and it’s long been observed that bears feed on salmon for several months despite the ephemerality of individual populations^19,20^. However, no study has linked this behavior to increased salmon consumption. We found that duration of salmon foraging explained 70% of the variation in the proportion of salmon in the assimilated diet and 49% of variation in the mass of salmon consumed. While it is intuitive that eating salmon for more days increases salmon consumption, numerous studies have shown that the consumption resulting from a day of fishing is highly variable due to variation in fishing ability^15,16^, salmon vulnerability^21,22^, salmon abundance^16^, intraspecific competition^15,16^, and bear reproductive status^23^. Thus, we find the explanatory power of foraging duration remarkable given the uncontrolled variation in our study. Bears that visited a single salmon spawning site foraged for an average of ~30 d^7^. This corresponds to the inflection point in our sigmoidal model of salmon consumption, where increasing foraging duration had the strongest effect on consumption. Because typical salmon sites only have spawning salmon for ~40 days, the strongest benefits of increasing foraging duration were achieved by visiting multiple spawning sites as bears surfed the red salmon wave.

Resource abundance generally is viewed as the key variable controlling consumption levels and food web dynamics^1^. Recent theoretical work has shown how another feature of food resources, phenological variation, can have a greater influence on consumption than food abundance^1^. However, no empirical studies have quantified these trophic variables in the wild despite clear significance to conservation and management^1,24,25^. In our study area, salmon abundance varied 2.5-fold between years, yet accounting for salmon abundance did not improve bear diet models. This suggests that in intact watersheds of coastal Alaska that have abundant salmon runs, interannual variation in salmon abundance likely has less effect on bear diets than individual variation in bear foraging behavior. Our results provide some of the first empirical evidence showing that phenological tracking can be a dominant driver of resource consumption. Human actions that reduce phenological diversity of salmon or interfere with bear movements (and thus their ability to track salmon phenology) likely have stronger effects on the bear-salmon trophic linkage than currently recognized. Consequently, management frameworks that use salmon escapement as a sole indicator of bear foraging opportunity likely fail to predict how bears respond to watershed alteration, salmon hatcheries or altered fishing policies.

We used two different tracer methods, mercury^26,27^ and C/N stable isotopes^28^, to estimate the contribution of salmon to bear diets. While mercury provides a direct measure of salmon consumption with minimal assumptions, the estimates of salmon in the assimilated diet by stable isotope analyses depend on multiple assumptions, several of which are not well understood. Nevertheless, we included stable isotope analysis in our research because it provided an independent corroboration and allowed us to place our results directly in the context of prior bear research that used stable isotope analyses^25,28–30^. Our stable isotope mixing space geometry (i.e., an oblique triangle) was suboptimal for comparing the contribution of plants versus deer, but was adequate for our central objective of estimating the dietary contribution of salmon (Supplementary Fig. 1). While there are encompassing theories to begin explaining variation in nitrogen discrimination^31^, we know of no such comprehensive theory for carbon discriminations that likely vary with diet, level of intake, season and animal physiology. Until this issue is resolved, many stable isotope analyses (including this one) have assumed that carbon discrimination is fixed across time and foods^23–36^. Despite the sources of uncertainty inherent to stable isotope analyses, percent salmon in assimilated diets was strongly related to estimates of salmon consumption derived from mercury analysis (saturating model, R^2^ = 0.69; Fig. 3a). This suggests that stable isotope analysis remains a useful tool and validates the many studies that have used stable isotope derived estimates of diet composition to make inferences about the salmon consumption of bears^25,28,37^ and other consumers^38^.

Larger bears both consumed more salmon and had more salmon dominated assimilated diets (Fig. 3b,c). These patterns are consistent with research showing that larger bears can exclude smaller bears from prime fishing locations^16^, and that larger bears have more difficulty gaining weight on dispersed, less nutrient dense foods^39–41^. We only studied female bears, but prior research found that male Kodiak bears (which tend to be larger) consumed ~2 times more salmon than females^30^.

Prior work^37^ relating salmon consumption to reproductive success (female litter size) suggests the increase in salmon consumption would increase litter size by 0.3 cubs, a roughly 14 percent increase in fecundity. So, given this benefit, why didn’t all female bears consume > 1500 kg of salmon/year? There are probably tradeoffs associated with salmon consumption. For example: (1) Bears may eschew salmon to avoid the risk of encountering other bears^23^ or humans^42^; (2) They may seek alternative foods that are more nutritious than salmon^43^; and (3) They may forage on less nutritious foods, such as low protein-high carbohydrate berries, that when combined with lesser amounts of high protein salmon allow rapid weight gain^41^. A remaining challenge is to understand the drivers of individual variation in phenological tracking, which our study shows is strongly related to salmon consumption.

It is widely assumed that the dense populations of bears in coastal Alaska are the result of abundant salmon. This study emphasizes the complementary importance of phenological diversity, which allows bears to feed on salmon for longer and more fully capitalize on this pulsed resource. To exploit phenological diversity, bears must move across landscapes to integrate across sequentially spawning salmon populations. Thus, salmon abundance, salmon diversity, and landscape connectivity interact synergistically to enhance the trophic linkage between bears and salmon. These key ingredients for bears are increasingly at risk due to human activities, such as hatchery salmon supplementation, which tends to reduce the salmon genetic diversity that underpins phenological variation^44^, and extractive industry development that reduces habitat connectivity^45^.

## Methods

### Study area

This work was completed in the Karluk Lake area of southwest Kodiak Island, Alaska. The study area has three primary river-lake systems with dozens of tributaries. Sockeye salmon are abundant and tend to spawn in shallow habitats where they are vulnerable to predation by bears^21,22^. The average sockeye run size in the Karluk Lake system over the last 56 years (after commercial harvest) was 549,507 ± 34,029 (standard error of the mean, s.e.m.). Pink salmon are also abundant in the study area (average run size = 491,375 ± 96,849 (s.e.m.)), but spawn primarily in main-stem rivers where they are less vulnerable to bear predation^22^.

### Hair sampling

To understand how salmon consumption varied as a function of foraging behavior, we quantified the salmon consumed by 19 radio-collared female bears with known amounts of time spent fishing for salmon. We used time spent on salmon streams as a proxy for salmon foraging duration because a prior study found that bears are rarely detected near streams and rivers when salmon are not present^7^. The stable isotope ratios (δ^15^N, δ^13^C)^28^ and mercury concentration^26,30^ in a bear’s hair reflect their assimilated diet composition and salmon consumption, respectively, during the period of hair growth.

We collected hair from 9 bears in June 2012 and 10 bears in June 2015 that were carrying GPS collars which were deployed the prior year. We directly sampled guard hairs from 14 of the bears. For the other five bears we could not recapture in 2015, we used recent (within 7 days) GPS locations to identify putative bed sites, which were indicated by clusters of observations with low activity values (activity is recorded by an accelerometer within each collar^46^) and visited them in early June to collect hair in the beds. We also collected hair from an additional 9 bears in June 2012 and 6 bears in June 2015 during captures. Unlike the other 19 bears, these bears did not wear collars during the previous year, and therefore did not have corresponding foraging and habitat use data from the previous summer. We used these additional samples to compare consumption estimates derived from stable isotopes (% salmon in assimilated diets) and mercury (kg salmon). We also used previously published data from 36 bears to compare salmon consumption estimates from % salmon in assimilated diets and mercury^30^. All capture procedures were performed in accordance with guidelines and regulations approved by the Fish and Wildlife Service Institutional Animal Care and Use Committee (IACUC permit # 2012008, 2015-001).

### Hair preparation and isotope ratio estimation

June is a transition period for hair growth in Kodiak bears in that new hair is beginning to grow but the previous year’s guard hairs are still present. The old hair coat is not completely shed until the new hair coat is well along in the growth process. Thus, guard hairs from the previous year’s hair coat can be collected either during handling or from bed sites, and salmon or other high-quality foods provide the protein and energy for the new hair growth. In our study area, salmon is generally the earliest available protein rich food^47^. The resulting hair growth period^26^ encompasses the period of salmon foraging, which occurs from early July through October^7^. We assumed that hair growth rates were consistent during the growing period.

Hair samples were washed with distilled water and a 2:1 chloroform: methanol mixture to remove debris and oils and then ground into a fine powder^26^. Isotope ratios for hair and foods were determined with a continuous flow isotope ratio mass spectrometer (Delta PlusXP, Thermo Finnigan) at the Washington State University Stable Isotope Core Laboratory. Nitrogen (δ^15^N) isotope ratios are reported as per mil (‰) relative to atmospheric nitrogen. Carbon (δ^13^C) isotope ratios are reported as per mil (‰) relative to Vienna Peedee Belemnite (VPDB) by assigning a value of 1.95‰ to NBS 19 calcium carbonate. Laboratory reference standards (acetanilide and keratin for nitrogen and B2155 casein and RM1547 for carbon) were interspersed throughout each analysis to ensure maintenance of calibration. The carbon standards had been previously calibrated to NBS 19, RM 8542, and IAEA-CO-9. Analytical errors (±1 SD) for the above standards were ≤ 0.1‰ for both nitrogen and carbon.

### Mercury estimation

The mercury contents of bear hair and their foods were determined at the University of Idaho Analytical Science Laboratory, Moscow, Idaho. Samples were digested in a solution of metal grade hydrochloric acid and nitric acid. The resulting mercury was reduced to elemental mercury vapor by stannous chloride in a continuous flow manifold. Ultra-high purity argon removed the mercury vapor in a gas-liquid separation chamber, which was then quantified by cold vapor atomic fluorescence^48^. The error in analyzing known standards was <2%.

### Diet composition estimates

Diets were estimated using the mixing model MixSIAR (version 3.1.7)^49^ in R^50^. The foods used in MixSIAR were black-tailed deer, salmon, and plant matter. Because of the difficulty in developing a plant matter value representative of the highly variable plant diets consumed by bears across such a large landscape, we subtracted isotopic discrimination values typical of ruminants (Δ^13^C: 2.7‰, Δ^15^N: 4.0‰)^51,52^ from black-tailed deer values to arrive at the plant matter endpoint and then used plant matter discrimination values from bears to arrive at the endpoint for bears foraging only on plant matter. The calculation of this endpoint incorporated variation around the bear discrimination values and variation around deer source values. This approach recognizes that both deer and bears are selective feeders on forbs and grasses in the spring and early summer, the period over which the plant matter is integrated into deer tissue. The source isotopic values used were: δ^15^N, 0.5 ± 0.5‰ for plant matter (n = 6) and 10.7 ± 0.4‰ for sockeye salmon (n = 10); δ^13^C, −26.6 ± 2.4‰ for plant matter (n = 6) and −21.8 ± 1.2‰ for sockeye salmon (n = 10). Nitrogen discriminations used were 5.2 ± 0.5‰ for plant matter and 3.6 ± 0.6‰ for sockeye salmon. We used a constant 3.7 ± 0.2‰ as the carbon discrimination for all foods^23–36^. Our models were structured with bear identification number as a random effect, process error, and uninformative priors. We evaluated models with and without concentration dependence and performed Gibbs sampling for each model using 3 chains, a burn-in of 500,000, and chain length of 1,000,000. Model diagnostics indicated satisfactory convergence of models. Although we explored the use of both concentration independent^28^ (e.g., assimilated diet) and concentration dependent^53^ (e.g., diet) models, we opted to use the concentration independent model because of (1) the enormous uncertainty in estimating the relative contribution and digestibility of the wide variety of plant matter consumed by bears in our study area across space and time^54^, (2) the lack of any improvement in the relationships between assimilated dietary salmon content and the other response variables when using concentration dependent models, and (3) the desire to compare to previous results on Kodiak and elsewhere that used concentration independent models^28,30,37^. For the benefit of others that may want to use concentration dependent models, we provide both sets of diet estimates in the supplementary material (Supplementary Table S1).

We also assessed a two-source model that excluded deer as a source. We did this because of some existing evidence suggesting deer is not a common food for bears in Kodiak^55^. In 2001, researchers conducted scat surveys in which 283 scats were collected from early June - early October. Scats were microscopically examined and corrected for differential disappearance to estimate % digestible dry matter of bear foods. The study found that 68% of digestible dry matter was from plant matter, 27% from salmon, 1.9% from ungulates, and 2.2% from other sources. Although these surveys found minimal evidence for consumption of deer, their sampling season did not encompass April and May when bears may have scavenged on winter-killed deer or killed and eaten deer fawns. We compared mixing models that did and did not include deer and found almost no difference in % assimilated diets from salmon (mean difference of 1.1%). We decided to include deer in the model because the existing fecal survey data may underrepresent the consumption of deer and estimates of % salmon in assimilated diets were robust to the inclusion of deer in the mixing model.

### Salmon intake estimates

Salmon intake was estimated as in a prior study^30^ based on the observed hair mercury content of each bear and the amount of mercury that would have been ingested to produce that hair mercury content^27^. However, rather than using a single estimate of bear mass, we used the estimated or measured mass of each captured bear. Masses were estimated by an experienced bear biologist in 2011, and bears were weighed using a digital scale in 2014 (overall mean = 174 kg, SD = 29 kg). We assumed sockeye salmon were the only significant source of mercury (119 ± 5 ppb on a 100% dry matter basis, 32.7 ± 1.4 ppb mercury on a fresh weight basis, and 27.5 ± 0.8% dry matter), because foods of terrestrial origin were previously shown to contain negligible amounts of mercury^30^ and the bears did not forage where they could access non-salmon marine resources.

One of the important assumptions in using mercury to estimate annual salmon intake by wild bears is that mercury consumed in one year is completely excreted by the following year. This assumption has been validated in captive bears^26^. To ensure that this assumption held in wild bears, we sampled 9 adult female bears in our study area in June 2015 (7 months after the last salmon foraging opportunity). The mercury content of the blood from those bears averaged 5.3 ± 2.8 ppb, which was not higher than levels seen in bears without salmon in their diets^30^. Thus, no correction was needed for mercury that was consumed in previous years and not fully excreted as virtually all mercury consumed in previous years was excreted prior to growth of the current year’s hair.

### Screening for contaminated hair samples

Although we visited putative bed sites soon after they were used by collared bears, hair collected from beds (5 of 19 bears) could have been deposited by non-target bears. To check for contamination, we examined the salmon consumption rate (kg salmon/day of salmon foraging) for each bear. We calculated consumption rate by dividing estimated salmon consumption by the duration of foraging (in days). Salmon consumption rate is constrained by assimilative capacity^56^, and thus, can be used to identify spurious values. Indeed, one bear foraged on salmon for 20 days, yet its hair sample (collected from a bed site) indicated salmon consumption of over 1700 kg. This would only be possible with a salmon consumption rate 3.4x higher than published maximum consumption rates^56^ (Supplementary Figure S2). We excluded this hair sample in the analysis because it was collected using a method more prone to contamination and it suggested an unrealistically high consumption rate. We cannot know whether the remaining four samples collected from bed sites were contaminated, however, model selection and the significance of model parameters were robust to the removal of all hair samples collected from bed sites.

### Statistical analyses

To quantify how assimilated diet composition (% salmon) and salmon consumption (kg salmon) varied with duration of salmon foraging, we fit simple linear, saturating, and sigmoidal linear models. From each model suite, we selected the model with the lowest AICc^17^ as the top model, and then used model diagnostics to test for violations of regression assumptions. We also tested for effects of salmon abundance (which varied between the two study years) and foraging duration on salmon consumption using multiple linear regression. Prior to multiple regression modelling we transformed duration using ln(duration + 50) to meet regression assumptions.

### Data availability

All data generated or analyzed during this study are included in this published article (and its Supplementary Information files).

## Electronic supplementary material

Supplementary Information
