# Supplementary material for: Phenological tracking associated with increased salmon consumption by brown bears
Source: Sci Rep. 2018 Jul 20;8:11008. doi: 10.1038/s41598-018-29425-3 (PMC6054687; doi:10.1038/s41598-018-29425-3)
Supplement: Supplementary file 1 — Supplementary Information [file 41598_2018_29425_MOESM1_ESM.doc]

**Supplement**

*Title:* Phenological tracking associated with increased salmon consumption by brown bears

*Authors*

William W. Deacy*^1,2^, Joy A. Erlenbach^3^, William B. Leacock^4^, Jack A. Stanford^2^, Charles T. Robbins^3,5^, and Jonathan B. Armstrong^1^

^*^Corresponding author (will.deacy@gmail.com)

^1^ Department of Fisheries and Wildlife, Oregon State University, Corvallis, OR, USA.

^2^ Flathead Lake Biological Station, University of Montana, Missoula, MT, USA.

^3^ School of the Environment, Washington State University, Pullman, WA, USA.

^4^ Kodiak National Wildlife Refuge, United States Fish and Wildlife Service, Kodiak, AK, USA.

^5^ School of Biological Sciences, Washington State University, Pullman, WA, USA.

Supplementary Materials

Supplementary Table 1. Assimilated diet estimates using a concentration-independent model and concentration-dependent model for brown bears on Kodiak Island, Alaska, 2011 and 2014. Models had animal identification number as a random effect and process error. Mean, 1 SD, Median, and 95% CI (credible interval) are % estimates from the population-level model. Range denotes the range of median % estimates among individual bears. When concentration dependence was used, we followed Hopkins et al. 2017, using digestible elemental concentration values averaged from table S2 (plant matter: C= 45 + 0, N= 5.84 + 3.70, n= 43; deer: C= 51.5 + 0 , N= 15.5 + 0.54, n= 4; trout: C= 54.8 + 0 , N= 11.65 + 4.12, n= 6, with the trout value being identical to the salmon value as calculated in a prior study^40^.

|  | Concentration-Independent | | | | |  | Concentration-Dependent | | | |  |
| --- | --- | --- | --- | --- | --- | --- | --- | --- | --- | --- | --- |
|  | Mean | SD | Median | 95% CI | | Range | Mean | SD | Median | 95% CI | Range |
| Deer | 9.4 | 7.1 | 7.8 | | 0.7-26.8 | 1.8, 10.1 | 9.1 | 7.1 | 7.4 | 0.8-27.8 | 1.7, 10.9 |
| Plant matter | 26.2 | 7.2 | 26.3 | | 12.2-40.0 | 3.8, 78.0 | 36.7 | 10.2 | 37.5 | 13.9-54.5 | 5.6, 87.5 |
| Salmon | 64.4 | 6.2 | 64.5 | | 52.0-76.0 | 13.3, 93.5 | 54.2 | 7.1 | 54.1 | 40.5-68.4 | 7.4, 91.6 |

Supplementary Table 2. The relative proportion of salmon in the assimilated diet of bears and their absolute intake of salmon as a function of duration of stream use model metrics. The models plotted in figure 2 are italicized.

| Response | Model name | Model form | Parameters (K) | AICc | ∆AICc |
| --- | --- | --- | --- | --- | --- |
| *kg salmon* | *saturating* | *y=1711-(1711*(0.98^x^))* | *2* | *267.21* | *0* |
| kg salmon | sigmoidal | y=1365/(1+ e^-0.065 * (x-32.8)^) | 3 | 267.86 | 0.65 |
| kg salmon | simple linear | y=11.48x+322.6 | 2 | 268.00 | 0.80 |
| *% salmon* | *sigmoidal* | *y=78.58/(1+ e^-0.042 * (x-7.99)^)* | *3* | *151.69* | *0* |
| % salmon | simple linear | y=0.68x+18.90 | 2 | 152.12 | 0.43 |
| % salmon | saturating | y=82.7-(82.7*(0.98^x^)) | 2 | 152.12 | 0.43 |

Supplementary Figure 1- Carbon and nitrogen isotope values for 34 hair samples collected from brown bears (*Ursus arctos middendorffi*) in Southwest Kodiak. Mean (± 1 SD) carbon and nitrogen isotope values for bears consuming black-tailed deer, sockeye salmon, and plant matter are plotted with the shaded area showing the isotopic mixing space.


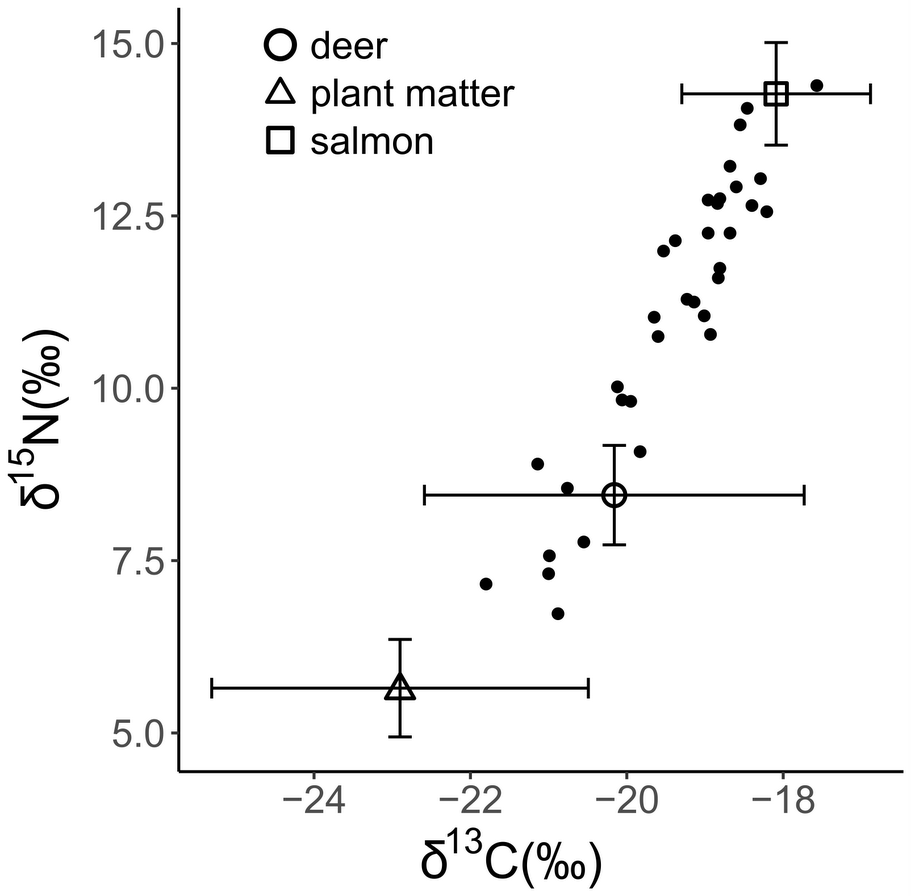


Supplementary Figure 2- Salmon consumption rates (kg fresh salmon/day). The vertical red line indicates the maximum foraging rate recorded for captive bears eating salmon (Hilderbrand et al. 1999). The outlier was not included in the analysis because it was collected from a bed site and it was likely hair from a non-target bear.


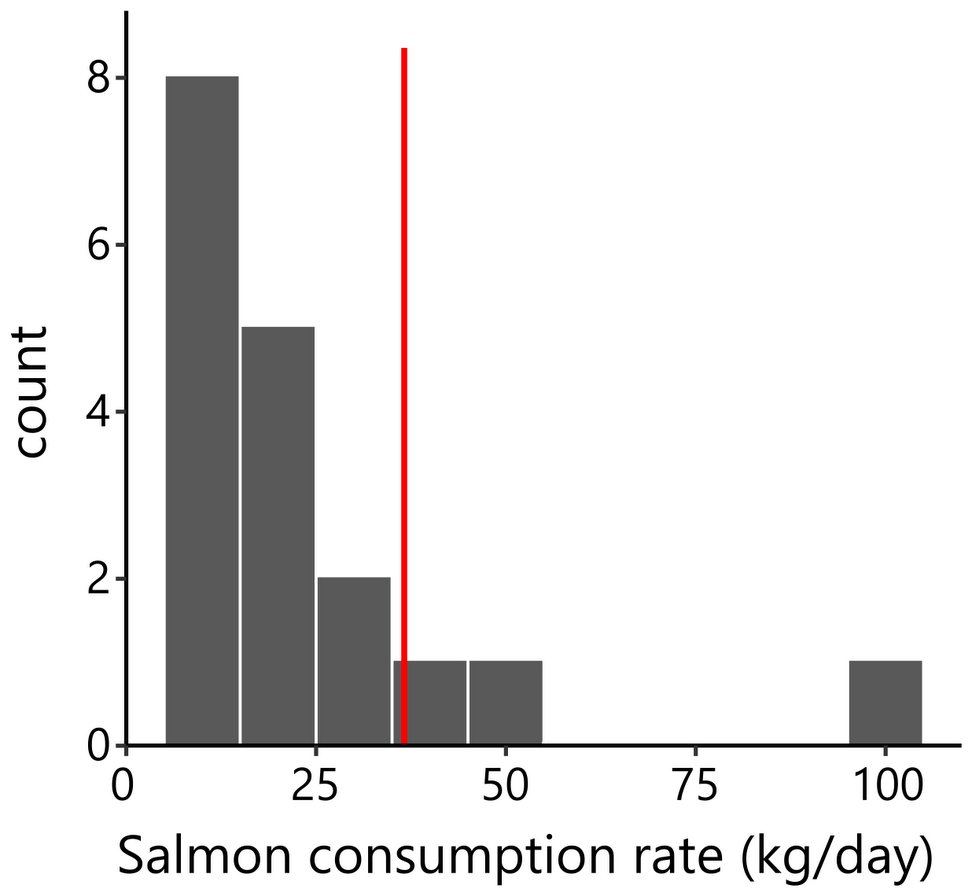


*Supplementary References*

Hilderbrand, G. V, S. G. Jenkins, C. C. Schwartz, T. A. Hanley, and C. T. Robbins. 1999. Effect of seasonal differences in dietary meat intake on changes in body mass and composition in wild and captive brown bears. Canadian Journal of Zoology 1630:1623–1630.

Supplementary Dataset 1- Individual assimilated diet estimates from concentration independent MixSIAR model.

|  |  | deer | | | | |  | vegetation | | | | |  | salmon | | | | |
| --- | --- | --- | --- | --- | --- | --- | --- | --- | --- | --- | --- | --- | --- | --- | --- | --- | --- | --- |
| id | year | mean | SD | median | lower95ci | upper95ci |  | mean | SD | median | lower95ci | upper95ci |  | mean | SD | median | lower95ci | upper95ci |
| K003 | 2015 | 0.133 | 0.168 | 0.067 | 0.002 | 0.66 |  | 0.69 | 0.145 | 0.719 | 0.284 | 0.883 |  | 0.178 | 0.07 | 0.179 | 0.038 | 0.311 |
| K004 | 2015 | 0.172 | 0.19 | 0.096 | 0.004 | 0.692 |  | 0.391 | 0.134 | 0.424 | 0.058 | 0.582 |  | 0.436 | 0.088 | 0.446 | 0.227 | 0.58 |
| K009 | 2015 | 0.13 | 0.129 | 0.084 | 0.003 | 0.457 |  | 0.244 | 0.096 | 0.254 | 0.033 | 0.404 |  | 0.627 | 0.081 | 0.631 | 0.452 | 0.779 |
| K011 | 2015 | 0.152 | 0.155 | 0.092 | 0.003 | 0.542 |  | 0.276 | 0.111 | 0.292 | 0.032 | 0.46 |  | 0.571 | 0.087 | 0.577 | 0.39 | 0.727 |
| K012 | 2015 | 0.142 | 0.139 | 0.093 | 0.003 | 0.506 |  | 0.254 | 0.1 | 0.267 | 0.036 | 0.427 |  | 0.603 | 0.083 | 0.608 | 0.426 | 0.752 |
| K013 | 2015 | 0.073 | 0.072 | 0.047 | 0.002 | 0.26 |  | 0.124 | 0.067 | 0.12 | 0.015 | 0.263 |  | 0.803 | 0.073 | 0.803 | 0.655 | 0.944 |
| K014 | 2015 | 0.078 | 0.078 | 0.051 | 0.002 | 0.286 |  | 0.124 | 0.065 | 0.12 | 0.013 | 0.255 |  | 0.798 | 0.078 | 0.799 | 0.639 | 0.941 |
| K015 | 2015 | 0.091 | 0.089 | 0.06 | 0.003 | 0.317 |  | 0.153 | 0.074 | 0.154 | 0.019 | 0.294 |  | 0.756 | 0.077 | 0.755 | 0.6 | 0.913 |
| K016 | 2015 | 0.06 | 0.061 | 0.038 | 0.002 | 0.222 |  | 0.1 | 0.057 | 0.096 | 0.01 | 0.222 |  | 0.84 | 0.069 | 0.841 | 0.7 | 0.966 |
| K017 | 2012 | 0.075 | 0.075 | 0.049 | 0.002 | 0.271 |  | 0.132 | 0.067 | 0.131 | 0.015 | 0.263 |  | 0.793 | 0.073 | 0.794 | 0.651 | 0.936 |
| K018 | 2012 | 0.037 | 0.039 | 0.024 | 0.001 | 0.142 |  | 0.061 | 0.041 | 0.054 | 0.004 | 0.16 |  | 0.902 | 0.052 | 0.905 | 0.789 | 0.984 |
| K019 | 2012 | 0.075 | 0.075 | 0.051 | 0.002 | 0.269 |  | 0.132 | 0.067 | 0.13 | 0.017 | 0.265 |  | 0.793 | 0.072 | 0.793 | 0.653 | 0.934 |
| K020 | 2012 | 0.172 | 0.191 | 0.093 | 0.003 | 0.686 |  | 0.389 | 0.134 | 0.421 | 0.054 | 0.58 |  | 0.439 | 0.09 | 0.449 | 0.227 | 0.585 |
| K021 | 2012 | 0.145 | 0.182 | 0.074 | 0.003 | 0.709 |  | 0.657 | 0.151 | 0.691 | 0.213 | 0.866 |  | 0.197 | 0.073 | 0.203 | 0.048 | 0.337 |
| K022 | 2012 | 0.096 | 0.095 | 0.064 | 0.002 | 0.344 |  | 0.185 | 0.082 | 0.192 | 0.027 | 0.328 |  | 0.719 | 0.074 | 0.719 | 0.579 | 0.874 |
| K023 | 2012 | 0.183 | 0.214 | 0.093 | 0.003 | 0.793 |  | 0.529 | 0.161 | 0.568 | 0.076 | 0.741 |  | 0.288 | 0.086 | 0.297 | 0.085 | 0.436 |
| K024 | 2012 | 0.191 | 0.215 | 0.101 | 0.003 | 0.765 |  | 0.455 | 0.153 | 0.495 | 0.063 | 0.663 |  | 0.354 | 0.093 | 0.365 | 0.132 | 0.507 |
| K025 | 2012 | 0.111 | 0.141 | 0.06 | 0.002 | 0.512 |  | 0.731 | 0.126 | 0.751 | 0.413 | 0.903 |  | 0.158 | 0.063 | 0.158 | 0.037 | 0.278 |
| K026 | 2012 | 0.072 | 0.071 | 0.048 | 0.002 | 0.259 |  | 0.126 | 0.065 | 0.123 | 0.013 | 0.253 |  | 0.802 | 0.074 | 0.802 | 0.662 | 0.948 |
| K027 | 2012 | 0.102 | 0.105 | 0.064 | 0.003 | 0.379 |  | 0.199 | 0.084 | 0.207 | 0.03 | 0.346 |  | 0.699 | 0.076 | 0.702 | 0.544 | 0.847 |
| K028 | 2012 | 0.182 | 0.216 | 0.089 | 0.003 | 0.802 |  | 0.502 | 0.159 | 0.546 | 0.066 | 0.71 |  | 0.316 | 0.089 | 0.328 | 0.095 | 0.46 |
| K029 | 2012 | 0.057 | 0.058 | 0.037 | 0.002 | 0.214 |  | 0.098 | 0.057 | 0.092 | 0.01 | 0.22 |  | 0.845 | 0.067 | 0.847 | 0.707 | 0.967 |
| K030 | 2012 | 0.137 | 0.141 | 0.084 | 0.003 | 0.505 |  | 0.278 | 0.105 | 0.295 | 0.039 | 0.446 |  | 0.585 | 0.079 | 0.589 | 0.415 | 0.734 |
| K031 | 2012 | 0.149 | 0.155 | 0.088 | 0.003 | 0.567 |  | 0.298 | 0.113 | 0.318 | 0.04 | 0.473 |  | 0.553 | 0.081 | 0.562 | 0.364 | 0.695 |
| K032 | 2012 | 0.168 | 0.182 | 0.099 | 0.003 | 0.662 |  | 0.373 | 0.128 | 0.402 | 0.055 | 0.559 |  | 0.459 | 0.088 | 0.468 | 0.25 | 0.607 |
| K033 | 2012 | 0.042 | 0.042 | 0.028 | 0.001 | 0.163 |  | 0.069 | 0.045 | 0.062 | 0.006 | 0.173 |  | 0.889 | 0.057 | 0.892 | 0.767 | 0.98 |
| K034 | 2012 | 0.158 | 0.196 | 0.08 | 0.002 | 0.752 |  | 0.625 | 0.158 | 0.66 | 0.169 | 0.835 |  | 0.217 | 0.079 | 0.223 | 0.045 | 0.355 |
| K036 | 2015 | 0.028 | 0.029 | 0.018 | 0.001 | 0.109 |  | 0.044 | 0.032 | 0.038 | 0.003 | 0.123 |  | 0.928 | 0.043 | 0.935 | 0.83 | 0.989 |
| K042 | 2015 | 0.11 | 0.107 | 0.074 | 0.003 | 0.389 |  | 0.198 | 0.085 | 0.203 | 0.027 | 0.35 |  | 0.692 | 0.079 | 0.693 | 0.533 | 0.848 |
| K043 | 2015 | 0.1 | 0.129 | 0.053 | 0.002 | 0.449 |  | 0.763 | 0.121 | 0.78 | 0.476 | 0.928 |  | 0.137 | 0.061 | 0.133 | 0.028 | 0.259 |
| K044 | 2015 | 0.133 | 0.133 | 0.085 | 0.003 | 0.48 |  | 0.244 | 0.099 | 0.255 | 0.032 | 0.408 |  | 0.623 | 0.083 | 0.625 | 0.448 | 0.781 |
| K045 | 2015 | 0.119 | 0.116 | 0.077 | 0.003 | 0.413 |  | 0.208 | 0.09 | 0.213 | 0.025 | 0.37 |  | 0.674 | 0.083 | 0.675 | 0.506 | 0.842 |
| K046 | 2015 | 0.065 | 0.066 | 0.042 | 0.002 | 0.24 |  | 0.111 | 0.062 | 0.106 | 0.011 | 0.243 |  | 0.824 | 0.071 | 0.826 | 0.68 | 0.954 |
| K047 | 2015 | 0.092 | 0.09 | 0.06 | 0.003 | 0.322 |  | 0.163 | 0.074 | 0.162 | 0.027 | 0.307 |  | 0.744 | 0.077 | 0.743 | 0.586 | 0.898 |
